# QM/MM Calculated Triplet–Singlet Infrared Difference Spectra of LOV2 Photoreceptor Domains and FMN in Solution

**DOI:** 10.1021/acsomega.6c06386

**Published:** 2026-08-18

**Authors:** D. P. Ngan Le, Gary Hastings, Samer Gozem

**Affiliations:** † Department of Chemistry, 1373 Georgia State University, Atlanta, Georgia 30303, United States; ‡ Department of Physics and Astronomy, Georgia State University, Atlanta, Georgia 30303, United States

## Abstract

Light-oxygen-voltage (LOV) domains are photoreceptors that mediate critical cellular responses to blue light in plants, fungi, and bacteria. In the LOV2 domain of phototropin 1 from *Avena sativa* (AsLOV2), the primary photophysical event following photoexcitation of the flavin mononucleotide (FMN) chromophore is intersystem crossing to the triplet state. This is followed by formation of other transient intermediates leading to an adduct that triggers protein conformational changes. It is possible to follow the formation and decay of those intermediates and conformational states using methods like time-resolved (TR) Fourier-transform infrared (FTIR) difference spectroscopy (DS). The interpretation of TR-FTIR DS can be aided by computational models that connect experimental signals with molecular modes that describe the chemistry of the system. In this work, a hybrid quantum mechanics/molecular mechanics (QM/MM) protocol is employed to calculate the triplet–singlet (T–S) TR-FTIR DS of FMN within AsLOV2 and for free FMN in water. For AsLOV2, snapshots from molecular dynamics (MD) simulations are selected to carry out QM/MM vibrational frequency calculations. Only a few MD calculated structures need to be sampled for the protein to produce (T–S) FTIR DS that are in good agreement with experiment. In comparison, considerably more sampling is required for flavin–water geometries to simulate the (T–S) FTIR DS of free FMN in solution due to the increased randomness of solvent interactions. The calculated spectra correctly capture the frequency shifts associated with the change in flavin’s electronic structure in the triplet state for both the protein and for free FMN in solution relative to the singlet. Deviations from the experimental frequencies may be explained in part by mixing between the lowest (π,π*) triplet state of FMN and the second (n,π*) triplet state, in accordance with recent reports in the literature of such mixing in free and protein-bound FMN.

## Graphical abstract

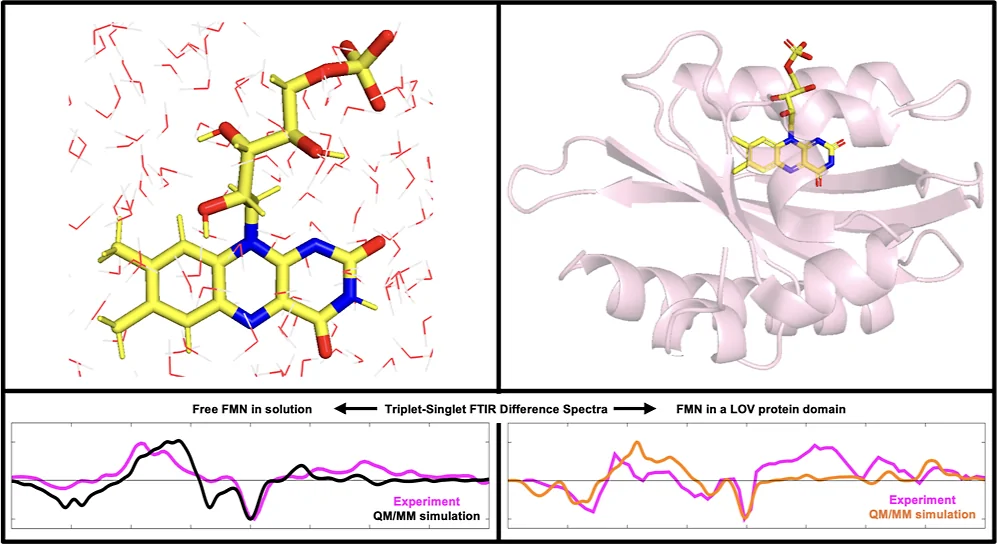

## 1 Introduction

Flavins play many roles in nature, earning them the reputation of “nature’s most versatile thespians.”^1^ Flavin mononucleotide (FMN) and flavin adenine dinucleotide (FAD) are redox cofactors in flavoenzymes where they catalyze a wide range of reactions.^2,3^ Beyond their redox functions, flavins also act as chromophores in several classes of photoreceptor proteins and photoenzymes, where they have pivotal roles in biological light sensing, transduction, and light-energy conversion.^4–9^ Specifically, in flavoprotein photoreceptors, flavins undergo photochemical transformations that couple light absorption to protein conformational changes and downstream signaling.^1,2,10^ In photoenzymes, flavins are increasingly being utilized to convert light energy into chemical energy by catalyzing thermodynamically uphill reactions.^11–15^

The (ultra)­fast time scales of photophysical and photochemical transformations in flavin photoreceptors and photoenzymes are probed by time-resolved (TR) techniques. TR Fourier-transform infrared (FTIR) difference spectroscopy (DS) is a particularly powerful means to study such transformations because it is sensitive to subtle changes in the pigment–protein interactions that occur upon excited-state formation.

Interpreting experimental FTIR DS may be aided by computational models that assign spectral bands to vibrational normal modes.^16–19^ However, benchmark studies of flavin in solution, in both oxidized and reduced states, have indicated that even subtle changes in the computational methodology and solute–solvent interaction strength may affect the agreement between simulated and experimental spectra.^19–22^ Simplified models do not always capture the frequencies and intensities of IR bands correctly, and may even predict incorrect ordering of vibrational frequencies, leading to incorrect assignments of experimental IR bands. Therefore, benchmarking of computational methods remains essential for developing protocols and workflows that are suitable for simulating FTIR signals.

Previous benchmarks for flavin in solution have focused on the diagnostic ∼1350–1750 cm^–1^ region, where prominent C=O and C=N bands appear. While implicit solvent models (e.g., a polarizable continuum model, PCM) can describe bulk solvation effects reasonably well, they fail to reproduce broadening effects and fine spectral details arising from specific H-bond interactions. Quantum mechanical/molecular mechanical (QM/MM) models on free flavin in solution revealed that the carbonyl C=O stretches are especially sensitive to the H-bonding interactions, and that their relative energies and broadening are best captured after adequately sampling of the flavin–water interactions.^19–21^ In contrast, the relative frequencies of the mixed C=N bands are less sensitive to environmental changes, but their intensities depend strongly on details of the computational model; experimentally, one C=N band is markedly more dominant than the other, while most computational models predict similar intensities unless long-range electrostatics (e.g., using PCM) are included.

Compared to gas phase and solution phase simulations, there are significantly fewer benchmark studies that have focused on simulating FTIR spectra of a cofactor like flavin when it is bound to a protein. Extending these simulations to the more heterogeneous protein environment practically necessitates the use of QM/MM models that provide an atomic-level description of the flavin–protein interactions. Several aspects of modeling FTIR DS spectra of flavin in proteins can be more challenging than modeling flavin in solution, as discussed in detail in ref 23. Furthermore, as discussed in ref 20, even minor deviations in computed frequencies or intensities can be amplified in difference spectra, which sometimes makes it a challenge to even qualitatively reproduce the main features of experimental FTIR DS. That said, at least one aspect of modeling flavin–protein interactions may be easier than modeling flavin–water interactions: hydrogen-bonding networks can be more static in a rigid protein scaffold than in solution, where the flavin and water are both free to rotate and interact in any orientation.

This work focuses on the LOV2 domain from *Avena sativa* phototropin 1 (AsLOV2), a system extensively characterized using FTIR spectroscopy and corresponding computations.^24–29^ Upon blue light excitation, FMN in AsLOV2 undergoes intersystem crossing (ISC) to a triplet state, followed by a putative proton-coupled electron transfer and subsequent covalent adduct formation with a nearby conserved cysteine residue.^8,30,31^ This photochemistry triggers conformational changes that propagate through the protein and controls downstream signaling pathways. TR-FTIR DS has contributed significantly to mechanistic understanding of triplet state formation, adduct formation, and protein conformational changes in this and other LOV domains.^24–29,32–102^ However, these insights have been derived almost exclusively from the experiments. When supporting calculations were included, they were often derived from gas phase or PCM flavin models.

Here, we focus on simulating difference spectra (DS) between the dark (singlet) and intermediate (triplet) states of FMN both in the AsLOV2 protein environment and in solution. Experimental triplet–singlet (T–S) FTIR DS are available for both.^24,32^ By comparing calculated and experimental DS, we can assess how well our models can capture the effect of the different environments on flavin’s FTIR spectra. If successful, such models can prove useful to study the effect of protein sequence variations or conformational changes on the FTIR signals of flavin.

## 2 Computational Methods

### 2.1 System Preparation

The initial coordinates for the LOV2 domain of *A. sativa* phototropin 1 were obtained from the Protein Data Bank (PDB ID: 2V1A).^103^ The structure, which is monomeric, includes FMN as the chromophore and spans residues 404–546 of the full phototropin sequence, encompassing the core Per-ARNT-Sim (PAS) LOV2 domain and flanking Jα helix relevant for light-induced signal transduction. The FMN chromophore is bound inside a well-defined pocket of the LOV2 domain, surrounded by several polar residues (Figure 1). Two glycerol molecules appearing in the PDB file were deleted, and missing hydrogen atoms were added using the LEaP module in AmberTools^104,105^ based on standard protonation states at pH 7.0. The FMN chromophore in its oxidized state was retained.

**Figure 1:**
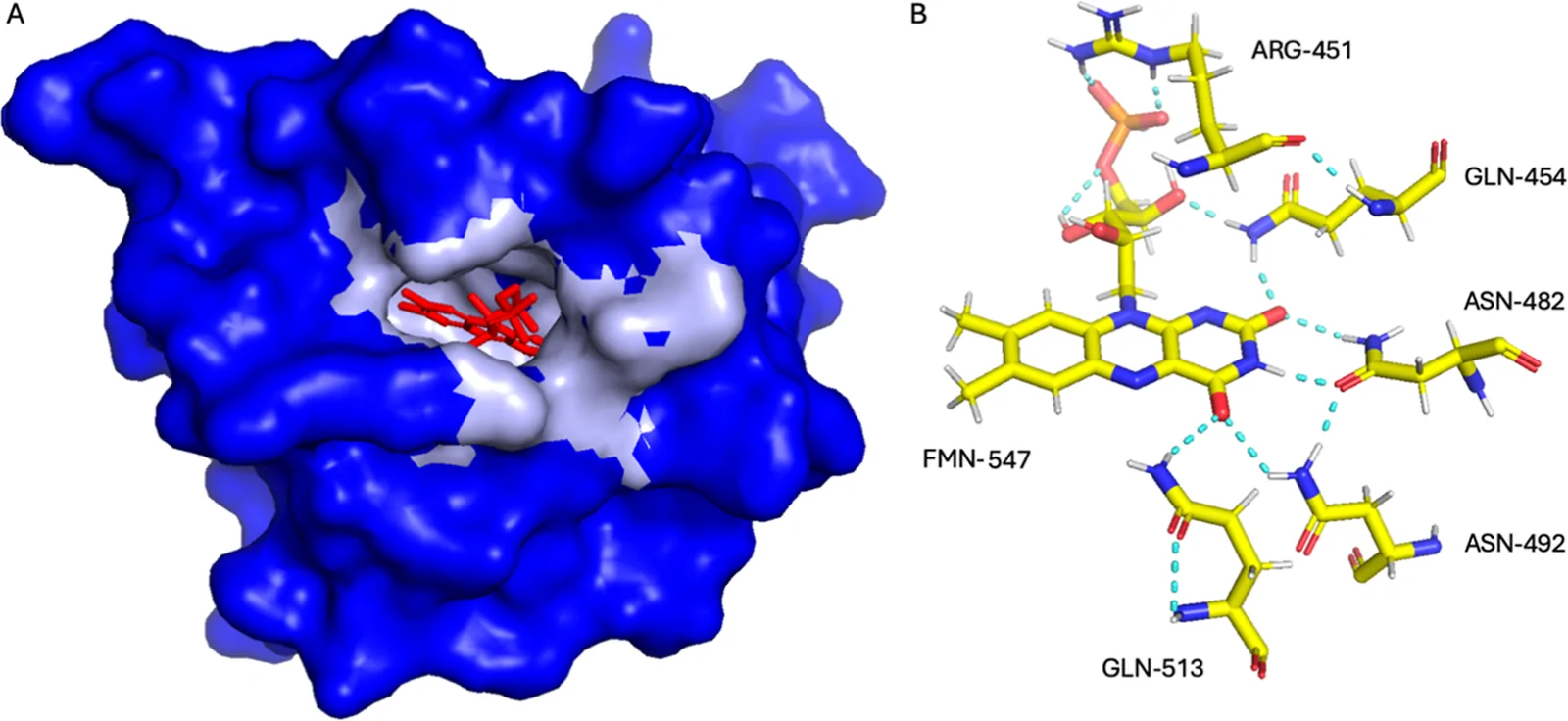
(A) Surface representation of the LOV2 domain showing the FMN chromophore (*red*) buried within the binding pocket (*light blue*) and surrounded by the rest of the protein (*dark blue*). (B) Close-up of the FMN binding site highlighting key hydrogen-bond interactions (*dashed lines*) with nearby amino acid residues.

### 2.2 Molecular Dynamics Simulations

Classical molecular dynamics (MD) simulations were performed using the Amber20 software suite^104,105^ to sample the conformational space of the LOV2 domain and to generate representative snapshots for subsequent QM/MM calculations. To prepare the system for MD simulations, the protein was solvated in a TIP3P^106^ cubic water box with a 10 Å distance between protein surface and the box edge. The overall negative charge of the protein was neutralized with sodium counterions (Na^+^). The protein was modeled using the AMBER ff19SB force field,^107^ with FMN parameters for the oxidized singlet state taken from our prior work.^20^ In the present study, FMN parameters for the triplet state were generated. Both singlet-state and triplet-state charges were parametrized using the RESP approach.^108^ Similar electrostatic potential fitting methods have been used to describe flavin as part of computational protocols to compute other electronic properties including UV/vis spectra, fluorescence, singlet–triplet energy gaps, and redox potentials.^109–116^

Following energy minimization to remove steric clashes from the initial structure, the system was equilibrated in two stages: first under constant volume and temperature (*NVT*) conditions for 500 ps to heat the system to 300 K, then under constant pressure and temperature (*NPT*, 1 atm) conditions to equilibrate the density. Production MD simulations were then carried out at 300 K for 5 ns, using periodic boundary conditions^117^ and a time step of 2 fs, with the SHAKE algorithm applied to constrain high-frequency bond vibrations.^118^ Snapshots were extracted every 10 ps from the equilibrated trajectory for further analysis.

For comparison, MD simulations of isolated FMN in aqueous solution FMN_(aq)_ were performed using the same protocol, except that the solvent box size was reduced to 3 Å from each dimension of flavin for consistency with earlier protocols, where testing showed a limited benefit of using a larger solvent box size for the solvated flavin.^19^ All other parameters, including temperature, time step, and flavin and solvent force fields, were identical to those used for the protein simulations.

### 2.3 QM/MM Optimization and Frequency Calculations

To model the electronic structure of the chromophore in the protein environment, we employed the ONIOM QM/MM approach^119^ as implemented in Gaussian 16 (Gaussian Inc., Connecticut).^120,121^ The QM region consisted exclusively of the FMN chromophore, treated at the B3LYP/6-31+G* level of theory for the singlet state and unrestricted B3LYP/6-31+G* for the triplet. The choice of functional and basis set is based on benchmarking carried out using a QM cluster model of flavin surrounded by water molecules.^22^ The MM region included the rest of the protein, solvent, and ions, where the protein was described by the Gaussian’s built-in Amber force field and water was described using TIP3P.^106^ Electrostatic embedding was used to capture the influence of the MM environment on the QM region.^122^ For each MD snapshot selected from the equilibrated singlet and triplet states of FMN, QM/MM geometry optimizations and harmonic frequency calculations were performed for the corresponding electronic state.

The protocol above is closely related to the QM/MM protocol labeled as M1 in ref 19. There, we found that the M1 protocol did not quantitatively capture the effect of water solvation on the flavin’s C=O vibrational frequencies nor for the relative intensities of the C=N bands. Capturing those effects for free flavin in water required the treatment of water molecules near the flavin hydrophilic side quantum mechanically, and accounting for long-range interactions using a hybrid QM/MM/PCM approach. However, in the protein environment, the relatively static, low-dielectric flavin binding pocket results in a stable hydrogen bond network that is not as dynamic as for free FMN in solution. Such an environment may be more amenable to the approximations used in a simple electrostatic-embedding QM/MM approach. This will be further discussed in the Results and Discussion sections.

In addition to the QM/MM simulations above, a few additional test models and calculations were carried out. First, we optimized FMN in the ground singlet and lowest energy triplet states and computed vibrational frequencies in an exclusively implicit PCM solvent with dielectric constant set to 78,^123,124^ providing a secondary reference for comparison with QM/MM-computed difference spectra in solution. Second, one snapshot of FMN in water and in AsLOV2 was selected to test the effect of truncating the ribose phosphate tail to replace it with a methyl group, yielding lumiflavin (LF). LF was optimized in the gas phase, PCM, and at the QM/MM level within the static MM environment of the solvent/protein. These calculations serve as a test for the effect of using LF as a model system instead of FMN. Third, the LF PCM model was also used to carry out TD-DFT optimization and frequency calculations for the second triplet excited state (T_2_). While the lowest (T_1_) triplet state has π,π* character (Figure 2 top), the T_2_ triplet state has n,π* character (Figure 2 bottom). Computational studies have suggested that while ISC from the singlet state to the triplet state may be driven by the n,π* triplet state, this ISC step is likely followed by rapid internal conversion to the lowest energy triplet T_1_ state having π,π* character.^113,114,125^ Therefore, the TR-FTIR triplet state signatures measured for flavin in both solvent and in AsLOV2 are expected to correspond to the lowest energy triplet π,π* state. However, it is useful to also simulate the n,π* state signatures, especially considering recent reports that indicate mixing in the electronic characters of the π,π* and n,π* states both in the singlet and triplet states in free FMN and in LOV proteins.^126,127^

**Figure 2:**
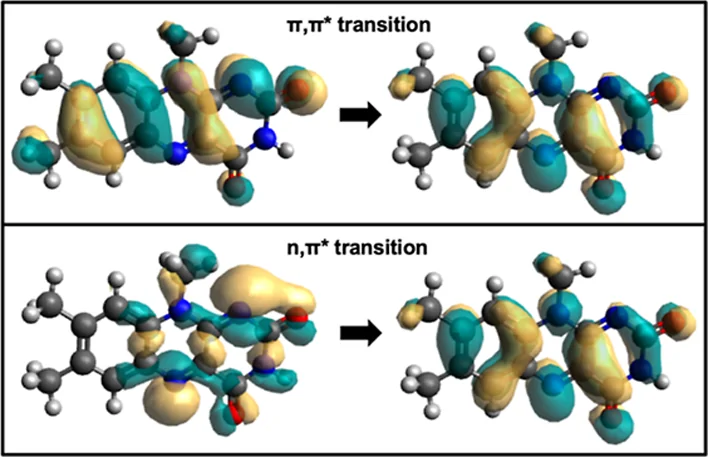
Molecular orbitals with the largest contributions to the lowest energy π,π* state and second lowest n,π* triplet state.

In previous work, it has been reported that the optimized geometry of the T_2_ n,π* state in flavin is very close to a state crossing with the T_1_ π,π* state.^113,114,128^ Indeed, TD-DFT optimization of the T_2_ n,π* state attempted in this work led to frequent root flipping and negative vibrational frequencies. The simulation of the vibrational features that would accurately capture the π,π* and n,π* state mixing, especially using ensemble QM/MM calculations in the solvent and protein, would likely require methods that accurately capture not only the relative energetics of these states, but also can correctly capture the crossing region and anharmonicity effects.^130^ Therefore, we limit the scope of our discussion here on TD-DFT calculations of the T_2_ n,π* state carried out for the minimal LF model in PCM.

### 2.4 FTIR DS Simulations

To simulate the (T–S) FTIR DS, 100 calculated spectra for both the singlet and triplet were used. For the singlet, the DS were scaled using empirically determined factors and broadened using Gaussian functions with a full-width at half-maximum (fwhm) of 8 cm^–1^. A frequency scaling of 0.969 (in solution) and 0.970 (in protein) were used to match the frequency of the experimental C=N_(mixed‑1)_ band at 1550 cm^–1^. This scaling factor is comparable to the recommended scaling factor for B3LYP reported in the NIST Computational Chemistry Comparison and Benchmark DataBase.^131^ Throughout this work, C=N_(mixed‑1)_ and C=N_(mixed‑2)_ designate the lower-frequency (∼1550 cm^–1^) and higher-frequency (∼1583 cm^–1^) C=N stretching modes of flavin, respectively. Both modes involve contributions from multiple internal coordinates, particularly C=N and C=C stretches as revealed by vibrational energy distribution analysis (VEDA). It is worth noting here that due to the highly mixed character of those bands, especially the one at ∼1583 cm^–1^, the literature has alternated between referring to those bands as C=C­(mixed) or C=N­(mixed). This is because the degree of mixing of C=C and C=N modes is sensitive to details of the solvent model, to the flavin model (LF vs FMN), and to other subtle changes in the computational approach, as discussed in prior work.^19^ In fact, this mixing is precisely why the relative intensities of the ∼1550 cm^–1^ and ∼1583 cm^–1^ vary from one model to another, as we will elaborate on later in this manuscript.

For the 100 snapshots of the triplet state, scaling factors of 0.978 (solution) and 0.980 (protein) were chosen, such that the calculated triplet bands closely follow the positions of the bands in experimental DS in the 1350–1750 cm^–1^ region. For the PCM calculations in solution, a 0.990 scaling factor was used for the singlet and 0.989 for the triplet. The DS was then obtained by subtracting the simulated singlet-state spectra from the triplet-state spectra for each computational protocol. The resulting difference spectra were plotted in the 1350–1750 cm^–1^ range which is where bands due to the key C=O and C=N stretching modes appear.

### 2.5 Vibrational Mode Assignment

VEDA was used to quantify the contribution of different internal coordinates for the PCM calculations of the singlet and triplet states of FMN.^132^ VEDA uses an internal-coordinate representation of the molecule to decompose each normal mode into contributions from stretching, bending, torsional, and out-of-plane motions.

Direct application of VEDA to the QM/MM calculations was not possible. Instead, for each spin state, a representative QM/MM snapshot whose calculated frequencies closely reproduce the ensemble-averaged QM/MM spectra was selected, and its vibrational features were assigned by comparison to the corresponding PCM-derived mode character. These analyses form the basis for band assignments in this manuscript.

## 3 Results and Discussion

Flavin’s π-conjugated isoalloxazine tricyclic ring gives flavin its distinct near-UV/visible, fluorescence, and redox properties. The same ring also gives rise to the prominent peaks in the IR DS in the 1350–1750 cm^–1^ range.^133^ Therefore, it is common practice in quantum chemical modeling of flavin FTIR spectra and DS to truncate the ribose phosphate “tail” in FMN (or FAD) and replace it with a methyl group to reduce the computational cost of the calculations. We have also modeled the FTIR spectrum of free flavin in water by using LF as a model for FMN and FAD in earlier work.^19,20^ To assess the validity of such an approximation, in Figure 3 we compare calculated FTIR absorption spectra of LF and FMN in the gas phase, implicit solvent (PCM), and in AsLOV2 (QM/MM). In the gas phase, pronounced spectral differences are observed between LF and FMN, with orange arrows across panels A and B highlighting the frequency shifts of major carbonyl and ring vibrations. The optimized structure of FMN in the gas phase still has the phosphate group in FMN pointing away from the isoalloxazine ring, so these frequency shifts are not associated with any H-bonding interactions between the ribose phosphate group and the isoalloxazine ring. Instead, the gas phase calculations suggest that a long-range electrostatic effect of the negatively charged (−2) phosphate is affecting isoalloxazine’s electronic structure. By the same logic, FAD would also differ spectrally from LF in the gas phase due to the negatively charged phosphate groups as well as possible stacking interactions between the adenine and isoalloxazine. When the FMN is instead embedded in a solvent dielectric environment with ε = 78, the simulated FTIR spectrum much more closely resembles that of LF (see orange arrows across panels **C** and **D**). This is because the electrostatic effect of the phosphate group is screened by the water solvent.

**Figure 3:**
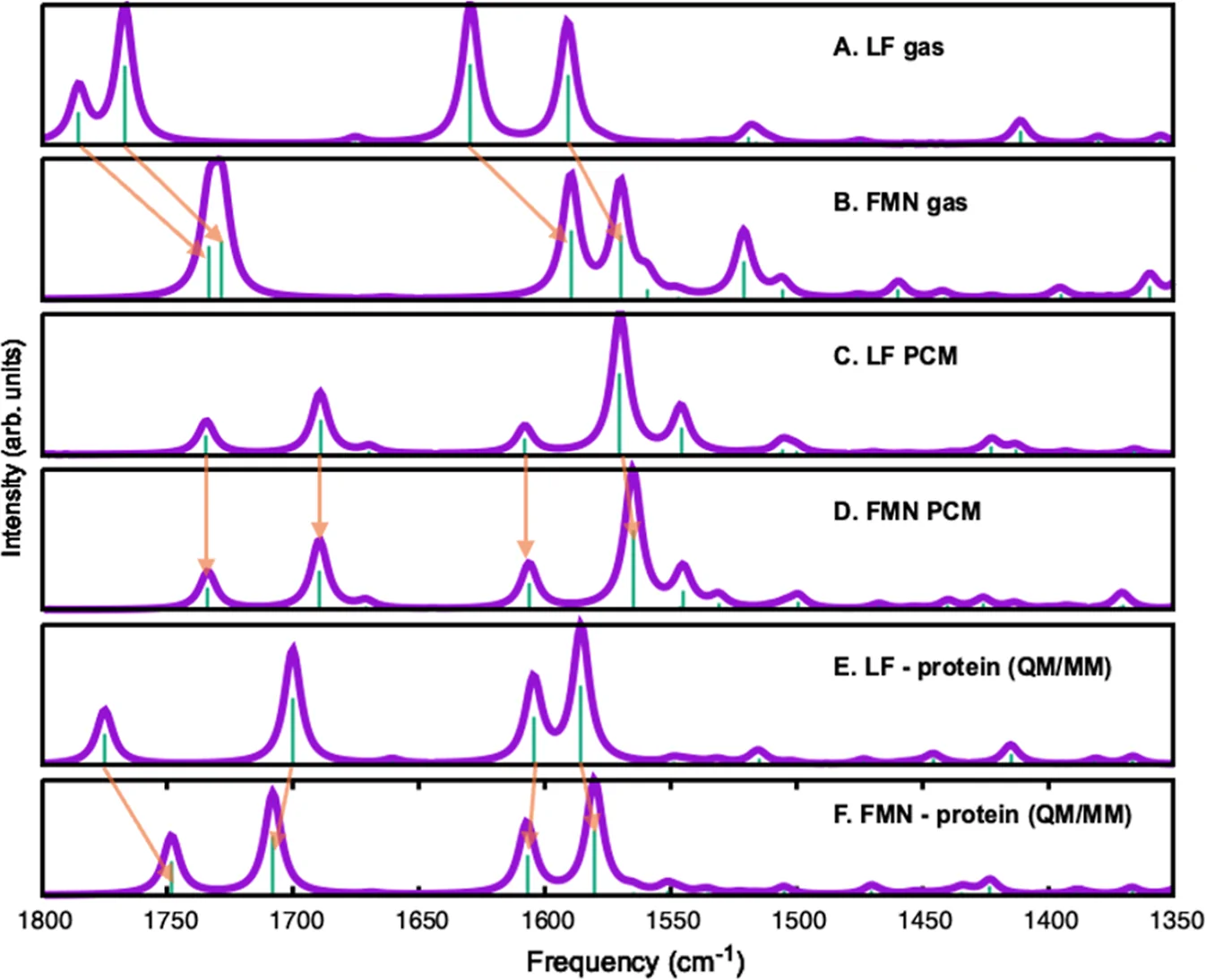
Simulated FTIR absorption spectra of LF and FMN in gas phase (A,B), implicit solvent (PCM, C,D), and protein environment (QM/MM, E,F). Spectra were computed at the B3LYP/6-31+G* level without applying scaling factors; hence, the frequency range was extended to 1800 cm^–1^. Orange arrows indicate key vibrational shifts of carbonyl and ring modes between LF and FMN. The lengths of key bonds from those models are included in the Supporting Information in Table S3.

In AsLOV2, the lower dielectric of the protein reestablishes electrostatic coupling between the phosphate and the flavin ring, resulting once again in non-negligible difference between FMN and LF (see orange arrows in panels **E** and **F**). These results indicate that while LF is a reasonable model system for simulating FTIR spectra of FMN in solution,^19,20,134^ it is not a good model in low dielectric environments like proteins because the electrostatic effect of the negatively charged phosphate group can no longer be ignored. For the remainder of this work, the full FMN model is included in the QM region.

Figure 4A,B show experimental and PCM calculated FMN (T–S) FTIR DS in aqueous solution, respectively. The PCM calculations, which are carried out here for the lowest triplet π,π* state and the singlet ground state, poorly reproduce the frequencies of the experimental FTIR bands in the higher-frequency region. The triplet calculation also poorly models the vibrational features appearing at lower frequencies associated with the triplet state. Since a constant empirical broadening factor is used, the different bandwidths are not captured well either.

**Figure 4:**
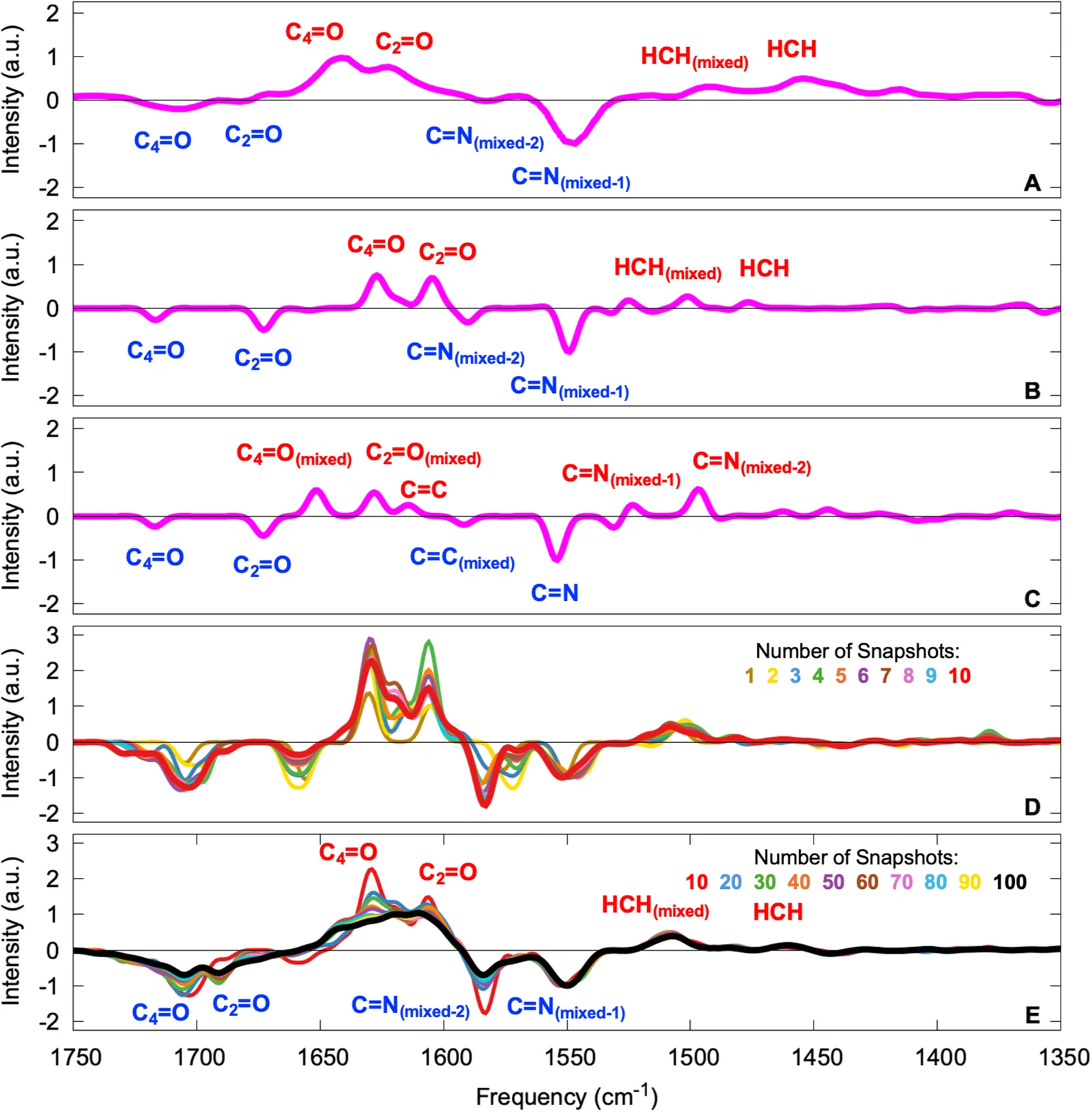
(A) Experimental (T–S) FTIR DS of FMN_(aq)_.^32^ (B) PCM-calculated (T–S) FTIR DS of FMN calculated for the lowest triplet state. (C) PCM-calculated FTIR DS of LF using the second (n,π*) triplet state instead of the lowest (π,π*) state. (D) QM/MM results of FMN_(aq)_ for summing 1–10 snapshots in single-integer increments and (E) 10 to 100 snapshots (in increments of 10). Band assignments shown are derived from VEDA analysis, where labels in red/blue correspond to positive/negative peaks associated with the triplet/singlet state, respectively. All simulated spectra are computed using the B3LYP functional and the 6-31+G* basis set. Triplet and singlet scaling factors of 0.978 and 0.969, respectively, were used for the QM/MM calculations while 0.989 and 0.990, respectively, were used for the PCM calculations. The corresponding frequencies and relative intensities are listed in Table 1.

Figure 4C shows the results of (T-S) FTIR DS simulation generated using the second (n,π*) triplet state, i.e., with frequencies calculated using TD-DFT instead of unrestricted DFT. The spectrum simulated using the n,π* triplet state is largely similar to the spectrum calculated with the π,π* state. However, there are a few notable differences. First, the C=O stretching bands are both shifted upfield in the n,π* state compared to the π,π* state shown in Figure 4B. The n,π* calculations in Figure 4C also present a small peak close to 1450 cm^–1^, which is consistent with the presence of an experimental peak near the same frequency. The π,π* calculation does not predict an allowed transition at that frequency. This, combined with the fact that the experimental C=O frequencies are in between those calculated using the π,π* and the n,π* triplet states, suggests that the experimental IR spectra have features of both the π,π* and n,π* states. This appears consistent with state mixing, as discussed in refs 126 and 127 .

Ensemble QM/MM models partially address the PCM model limitations. Figure 4D shows the effect of averaging up to 10 snapshots using the QM/MM modeling approach, which is clearly not sufficient to represent the ensemble features. Figure 4E shows spectra calculated using more extensive averaging of spectra for up to 100 MD snapshots, which converge to a spectrum that more closely resembles the experimental one. Overall, the QM/MM geometries give an improved agreement between calculated and experimental spectra, though convergence is relatively slow, especially in the high-frequency region where spectra obtained from 50 snapshots still compare poorly to experiment. This slow convergence reflects the large heterogeneity of H-bonding configurations in water, most clearly affecting the C=O stretching bands. The composite spectrum obtained by summing 100 QM/MM calculations (black line in Figure 4E) reveals that vibrational frequencies from individual calculations aggregate into several prominent bands that generally align well with experiment. However, discrepancies remain between experimental and calculated band intensities. In particular, the intensities of the negative features at 1550 cm^–1^ and above 1583 cm^–1^, that are associated with C=N_(mixed)_ stretches in the singlet state, are not well reproduced. Furthermore, the frequencies and the intensities of the singlet C=O stretching bands (negative signals at frequencies above 1650 cm^–1^) are not well reproduced either. These inaccuracies in the calculated intensities of the negative bands (belonging to the singlet state) were discussed in detail in ref 19 where the relative intensities of C=N bands could only be reproduced by using a more sophisticated model that includes the H-bonding waters explicitly in the QM region and that accounts for long-range solvation using a long-range PCM. Here, such a protocol is not pursued since an expanded QM/MM/PCM cannot be performed for the protein; we focus instead on comparing simulations for FMN in AsLOV2 vs. free FMN in water using a consistent protocol.

For the triplet vibrations appearing as positive bands in the DS spectrum, we find that the C=O frequencies are still slightly downfield compared to the experiment and that the calculations still do not reproduce the experiment peak close to 1450 cm^–1^. Again, these observations appear consistent with n,π* character mixing with the lowest π,π* state, and which the QM/MM calculations do not capture here.

Figure 5 shows experimental (Top) and calculated FMN (T–S) FTIR DS for AsLOV2. The same QM/MM protocol as applied for the bulk solvent was used for the protein. Figure 5B shows averaged spectra from up to 10 MD snapshots of protein-bound FMN, while Figure 5C shows the averaged spectra from 10 to 100 snapshots.

**Figure 5:**
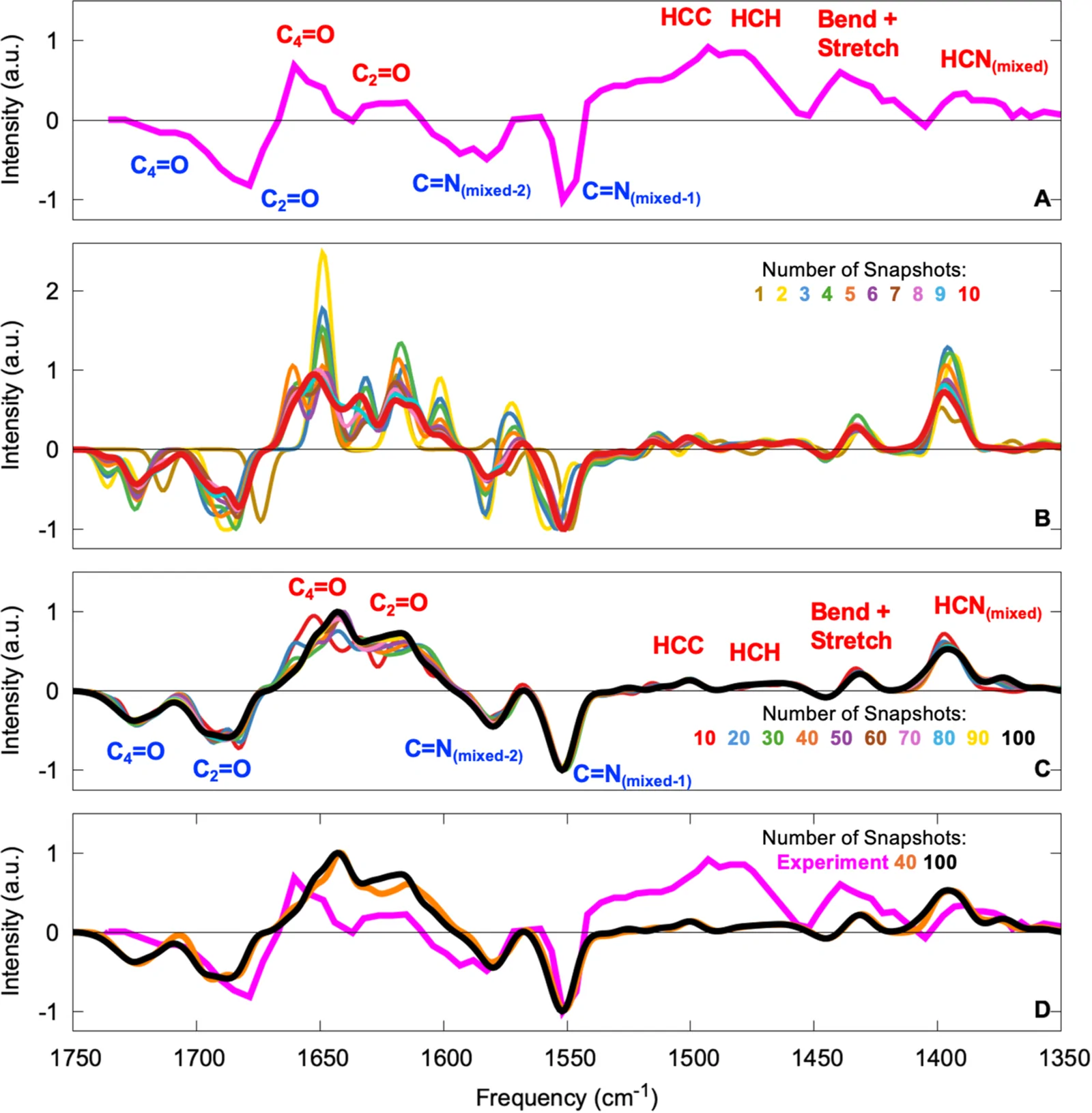
Comparison of AsLOV2 experimental (A) and calculated (B) (T–S) FTIR DS. Experimental DS is from ref 24. QM/MM calculated FTIR DS for summing 1 to 10 snapshots (B) and 10–100 snapshots (C). (D) Direct overlay of the experimental DS with QM/MM calculated DS summed over 40 and 100 snapshots, illustrating convergence of the calculated spectrum and its correspondence with experiment. Band assignments are indicated. Red/blue labels are assigned using VEDA and correspond to the triplet/singlet state, respectively. The “Bend + Stretch” label in red denotes a mode with mixed character, primarily involving NCC, CNC, and HCH bending coupled to C=C stretching. Simulated spectra are computed at the B3LYP/6-31+G* level with scaling factors of 0.970 (singlet) and 0.980 (triplet). The corresponding frequencies and relative intensities are listed in Table 1.

There are several important differences between the AsLOV2 calculated results compared to the solution results. First, the computed spectra for the protein converge more rapidly, requiring approximately 40 QM/MM snapshots. This is best highlighted in Figure 5D, where the spectrum obtained from 40 snapshots (orange) is overlaid on the spectrum obtained from 100 snapshots (black). This faster convergence relative to free FMN in solution is likely due to the more rigid (static) environment in the protein, especially with regard to hydrogen-bonding interactions where polar and charged residues form stable, directional interactions with FMN in the protein. These interactions reduce conformational flexibility and hence the number of microenvironments sampled during MD simulations. To demonstrate this, we present in Figure 6 an analysis of the average number of hydrogen bonding interactions formed between the carbonyl O_2_ and O_4_ atoms with the surrounding solvent (left) and protein (right) environments. The structures simply show a representative snapshot, but the labels indicate an average number of H-bonds and standard deviation calculated over 500 frames of the classical MD simulations using Amber’s CPPTRAJ tool.^135^ We find two relevant trends in the analysis: first, the number of H-bonding interactions invariably increase in the triplet state compared to the singlet state for both free FMN in solution and for AsLOV2. This indicates an increase in the polarity of the C=O bonds upon formation of the triplet state. Second, the standard deviation for the number of hydrogen bonds formed during the molecular dynamics is smaller in AsLOV2 than in water. This explains the faster convergence of the FTIR spectra, which requires fewer snapshots in AsLOV2 than in solution.

**Figure 6:**
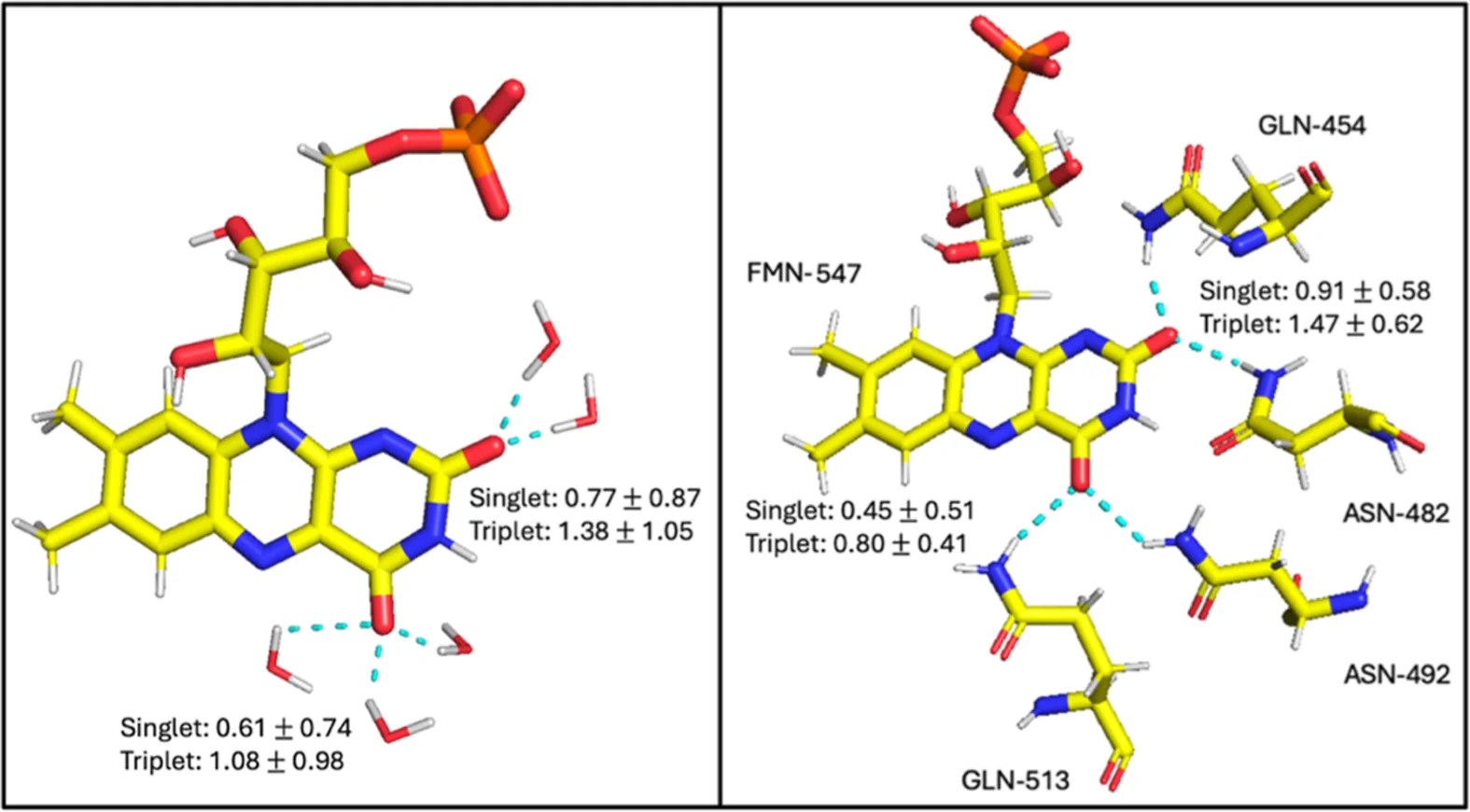
A randomly selected snapshot from the molecular dynamics of FMN in water (left) and in AsLOV2 (right), with nearby H-bonding residues shown. The labels indicate the average and standard deviation of hydrogen-bonds formed during the production stage of the molecular dynamics between the flavin C_2_=O_2_ and C_4_=O_4_ carbonyls and all surrounding water molecules/amino acids. Note that the number of H-bonds calculated here using CPPTRAJ may deviate from numbers reported in previous studies, e.g. for flavin in solution,^116,136^ due to a different criterion used by CPPTRAJ for defining H-bonds (3.0 Å distance, 135° angle).

Figure 5D overlays the simulated spectrum with the experimental one. The close correspondence with the experimental spectrum demonstrates that the QM/MM protocol—despite its limitations in bulk solvent discussed earlier—successfully captures the key spectral features of protein-bound FMN in AsLOV2. Interestingly, deviations appear again to be centered around the frequency of the carbonyl groups and around the intensity of bands near the 1400–1550 cm^–1^ region, which are expected to be enhanced if accounting for n,π* character, according to the PCM results in Figure 4.

Comparing Figures 4 and 5 reveals the sensitivity of band intensities to the electrostatic environment. For free FMN in solution, the singlet C=N_(mixed‑1)_ band is experimentally ∼5 times more intense than C=N_(mixed‑2)_ band. In AsLOV2, C=N_(mixed‑1)_ is instead around 2–3 times more intense than the C=N_(mixed‑2)_ band. Interestingly, the QM/MM protocol (in the absence of long-range electrostatic corrections) fails to reproduce this difference in intensity for FMN in solution—consistent with our earlier findings^19^—but captures it reasonably well in the protein environment. The fact that the relative intensities of the C=N_(mixed‑1)_ and C=N_(mixed‑2)_ bands are sensitive to the environment, both in experiment and in theory, is further evidence that the intensities are modulated by subtle changes in the C=C and C=N mode mixing that manifest as changes in intensity.^19^ This is also why the C=N_(mixed‑2)_ band is sometimes referred to as C=C in the literature.

A second difference revealed by comparing Figures 4 and 5 is a positive triplet band in the range of 1400–1450 cm^–1^, assigned to a mode with mixed NCC/CNC/HCH bending and C=C stretching character. This mode is experimentally observed in AsLOV2, but not for free FMN in solution. The QM/MM calculations correctly reproduce this environmental dependence.

Table 1 compares experimental band frequencies and normalized intensities with PCM and QM/MM calculations for key singlet and triplet features of FMN in aqueous solution and in AsLOV2. Band intensities are normalized relative to the singlet C=N band of S_0_, which is set to −1.0 in each difference spectrum. From visual inspection, the AsLOV2 experimental DS is better reproduced by the QM/MM calculations than FMN in solution, especially in the mid- and low-frequency region below ∼1550 cm^–1^ where protein-specific bending and mixed modes become prominent.

**Table 1:** Band Frequencies (in cm^–1^) and Normalized Intensities (in Parentheses, Normalized Relative to the Singlet C=N Stretching Mode Intensity) for key Singlet and Triplet FTIR Features of FMN in Aqueous Solution and in the AsLOV2 Protein^a^

band	FMN_(aq)_, exp	FMN_(aq)_, PCM	FMN_(aq)_, QM/MM	AsLOV2, exp	AsLOV2, QM/MM
singlet C_4_=O stretch	1709 (−0.21)	1717 (−0.27)	1705 (−0.70)	1715 (−0.16)	1725 (−0.38)
singlet C_2_=O stretch	1682 (−0.04)	1673 (−0.50)	1691 (−0.65)	1679 (−0.82)	1688 (−0.59)
singlet C=N_(mixed‑2)_ stretch	1583 (−0.03)	1590 (−0.33)	1584 (−0.70)	1583 (−0.49)	1580 (−0.45)
singlet C=N_(mixed‑1)_ stretch	1547 (−1.00)	1549 (−1.00)	1550 (−1.00)	1552 (−1.00)	1552 (−1.00)
triplet C_4_=O stretch	1642 (0.97)	1627 (0.74)	1619 (1.00)	1660 (0.69)	1643 (1.00)
triplet C_2_=O stretch	1622 (0.76)	1605 (0.68)	1610 (1.03)	1615 (0.22)	1617 (0.73)
triplet HCC bend	-	-	-	1493 (0.92)	1501 (0.13)
triplet HCH bend (mixed)	1494 (0.30)	1501 (0.26)	1507 (0.39)	-	-
triplet HCH bend	1455 (0.49)	1477 (0.13)	1461 (0.13)	1475 (0.77)	1464 (0.10)
triplet NCC, CNC, HCH bend and C=C stretch	-	-	-	1436 (0.54)	1432 (0.22)
triplet HCN bend (mixed)	-	-	-	1389 (0.34)	1396 (0.53)

a Experimental and QM/MM values are reported for the band maximum. Band assignments are based on vibrational energy distribution analysis (VEDA) performed on PCM-optimized geometries and are compared with QM/MM representative snapshots, selected as those whose computed frequencies best reproduce the ensemble-averaged QM/MM spectra for each spin state (see Vibrational Mode Assignment in Computational Methods). VEDA decomposition percentages for dominant internal-coordinate contributions to each mode, along with visualizations of representative normal mode vectors, are provided in the Supporting Information.

Calculations for FMN in solution and in the protein both capture the relative energetics of the C_4_=O and C_2_=O bands for the singlet (negative bands) and triplet (positive bands) states. For the singlet state, the experimental C_4_=O and C_2_=O bands in AsLOV2 are found at 1715 and 1679 cm^–1^, respectively, while QM/MM predicts these features at 1725 and 1688 cm^–1^, capturing the ordering and relative separation of the two carbonyl modes. Similarly, in the triplet state, the experimental C_4_=O and C_2_=O bands in AsLOV2 are observed at 1660 and 1615 cm^–1^ and are reasonably well reproduced by QM/MM calculations, at 1643 and 1617 cm^–1^, respectively. Again, the downfield shift of the computed bands relative to the experiment may be due to n,π* character mixing.^126,127^ Note that gas-phase CIS calculations on lumiflavin^24^ indicate that the C_2_=O band is at a higher rather than lower frequency than the C_4_=O band. This suggests that the solvent and protein environments invert the energetic ordering of these C=O bands compared to the gas phase.

QM/MM calculations generally capture the correct trend in relative intensities of the C=O bands, but do not quantitatively capture the relative magnitudes of those intensities in all cases. Nonetheless, the overall intensity pattern is substantially improved relative to solution-phase PCM calculations.

A comparison of the singlet and triplet bands reveals that excitation to the triplet state results in a systematic downfield shift of both the C_2_=O and C_4_=O bands in both solution and protein. For instance, in AsLOV2, the C_4_=O band of the singlet state downshifts 55 cm^–1^ from 1715 cm^–1^ to 1660 cm^–1^ upon triplet state formation. Similarly, in AsLOV2, the C_2_=O band downshifts 64 cm^–1^ (from 1679 to 1615 cm^–1^) upon triplet state formation. Comparable downshifts are also observed for FMN in solution, with both C=O modes downshifting 60 ± 10 cm^–1^. These shifts are well reproduced by the QM/MM calculations, indicating that the method reliably captures the electronic reorganization within the flavin moiety upon triplet-state formation.

In AsLOV2, the intense C=N stretching bands near 1580 and 1550 cm^–1^ diminish upon triplet formation and become strongly mixed with HCH and HCN bending motions. This mixing gives rise to several new triplet bands between 1500 and 1380 cm^–1^, including HCC, HCH, NCC, CNC, and HCN bending modes, which are absent or weak in solution-phase FMN spectra. These spectral changes are all consistent with a weakening of the π-conjugated bonding character across the flavin isoalloxazine ring due to triplet excitation, which redistributes vibrational intensity from localized stretching modes to mixed stretching-bending motions.

## 4 Conclusions and Future Directions

We simulated the (T–S) FTIR DS associated with FMN in solution and in the AsLOV2 protein binding site and found that an electrostatic-embedding QM/MM model can qualitatively capture the main features of the experimental (T–S) FTIR DS for FMN in both environments. In AsLOV2, sampling as few as 40 snapshots from MD simulations is adequate to obtain a converged spectrum, considerably fewer than the ∼100 snapshots needed for FMN in solution. This difference in convergence arises from the more heterogeneous water-FMN interaction orientations in solution. This conclusion can likely be extended to other similarly rigid proteins which undergo limited local dynamics around the FMN molecule and no extensive secondary structure changes.

While the QM/MM calculations qualitatively capture the relative frequencies and intensities of (T–S) FTIR difference bands, a more quantitative agreement will likely require improvements to the hybrid QM/MM methods used here. This may include a way to incorporate n,π* character mixing, as well as a better description of the electrostatic and hydrogen-bonding interactions in the FMN binding site either by using a larger QM space that includes the binding site amino acids or by using polarizable force fields.^137–139^ The use of QM/MM/PCM to capture long-range electrostatics may also be beneficial, as demonstrated for flavin in solution,^19^ but its routine application would require the use of algorithms to improve efficiency and convergence.^140,141^ The routine testing and validation of such methods against increasingly diverse experimental data sets will further enhance predictive power.

## Supplementary Material



## Data Availability

The data supporting this article have been included as part of the Supporting Information.

## Notes

Normal modes for the triplet and singlet state of FMN in PCM water model, corresponding VEDA results, and optimized xyz coordinates of LF and FMN in the singlet and triplet states in the gas-phase and PCM (PDF)
