# Supplementary material for: QM/MM Calculated Triplet–Singlet Infrared Difference Spectra of LOV2 Photoreceptor Domains and FMN in Solution
Source: ACS Omega. 2026 Aug 18;11(34):51851–63. doi: 10.1021/acsomega.6c06386 (PMC13625131; doi:10.1021/acsomega.6c06386)
Supplement: Supplementary file 1 [file ao6c06386_si_001.pdf]

Supporting information for:

QM/MM Calculated Triplet − Singlet Infrared Difference Spectra of LOV2 Photoreceptor Domains and FMN in Solution

D.P. Ngan Le,[†] Gary Hastings,*[,§,†] Samer Gozem*[,†]

[†] Department of Chemistry, Georgia State University, Atlanta, Georgia 30303, United States
[§] Department of Physics and Astronomy, Georgia State University, Atlanta, Georgia 30303, United States

Email: ghastings@gsu.edu, sgozem@gsu.edu

Table of content

Figure S1: Normal modes for triplet FMN (π,π*) in PCM water model.

Figure S2: Normal modes for singlet FMN in PCM water model.

Figure S3: Normal modes for triplet LF (n,π*) in PCM water model.

Table S1: VEDA results for triplet FMN (π,π*) in PCM water model.

Table S2: VEDA results for singlet FMN in PCM water model.

Table S3: Selected bond lengths (Å) for optimized LF and FMN in different models and states.

Table S4: VEDA results for triplet LF (n,π*) in PCM water model.

XYZ coordinates of optimized LF and FMN structures in singlet and triplet states under gas-phase and PCM conditions.

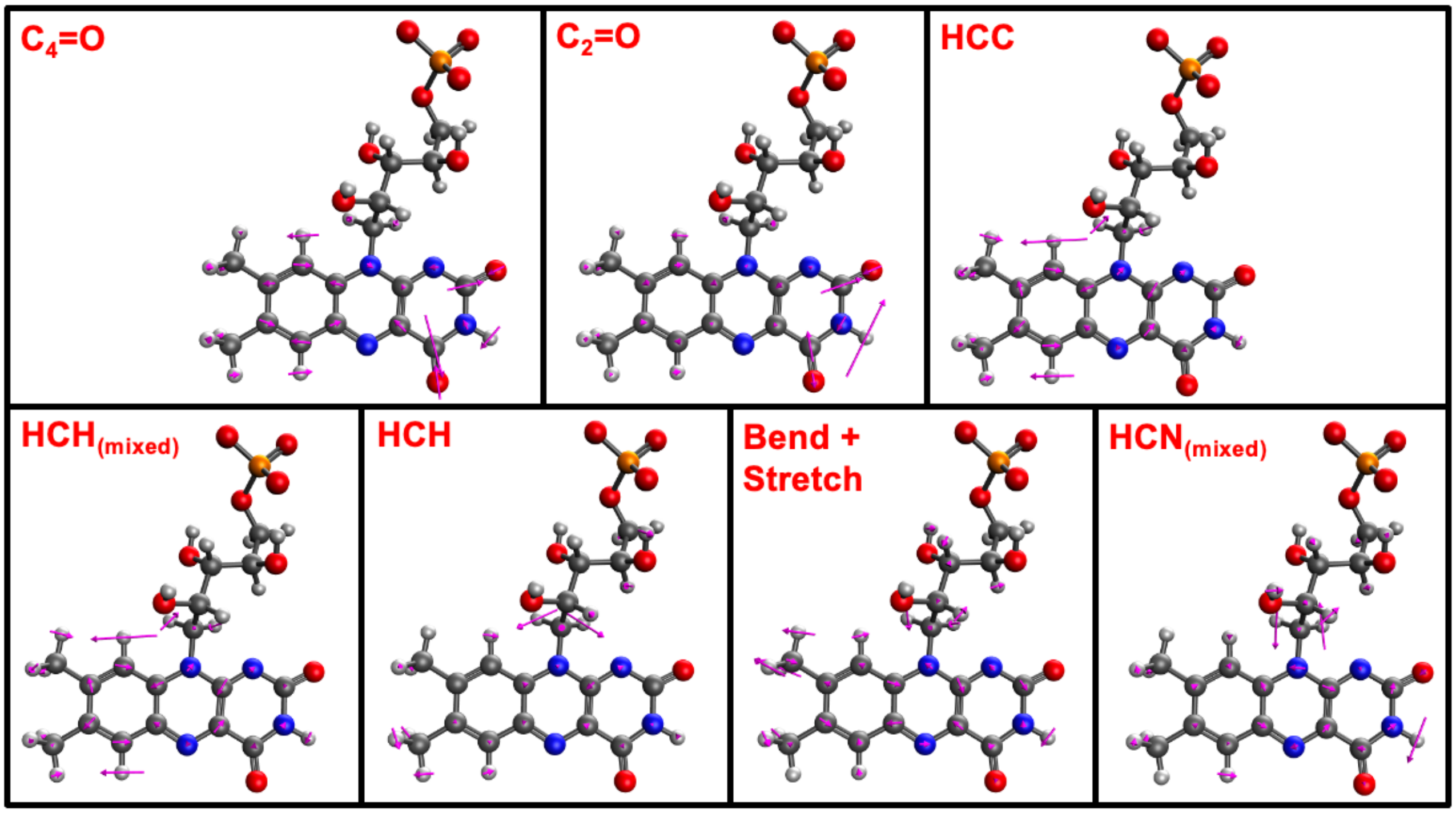

Figure S1: Normal modes computed with the B3LYP/6-31+G*/IEF-PCM model for triplet FMN (π,π*).

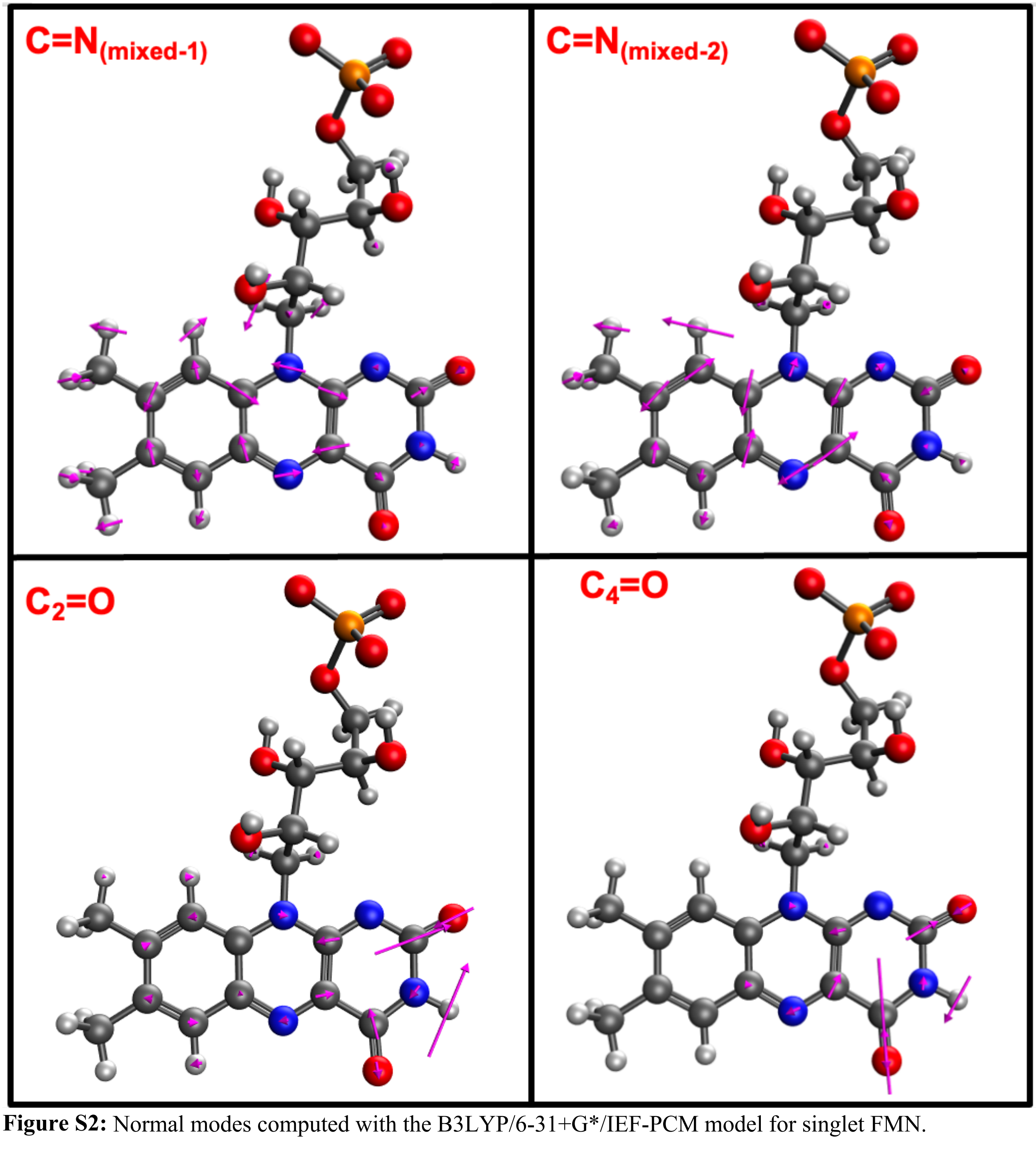

Figure S2: Normal modes computed with the B3LYP/6-31+G*/IEF-PCM model for singlet FMN.

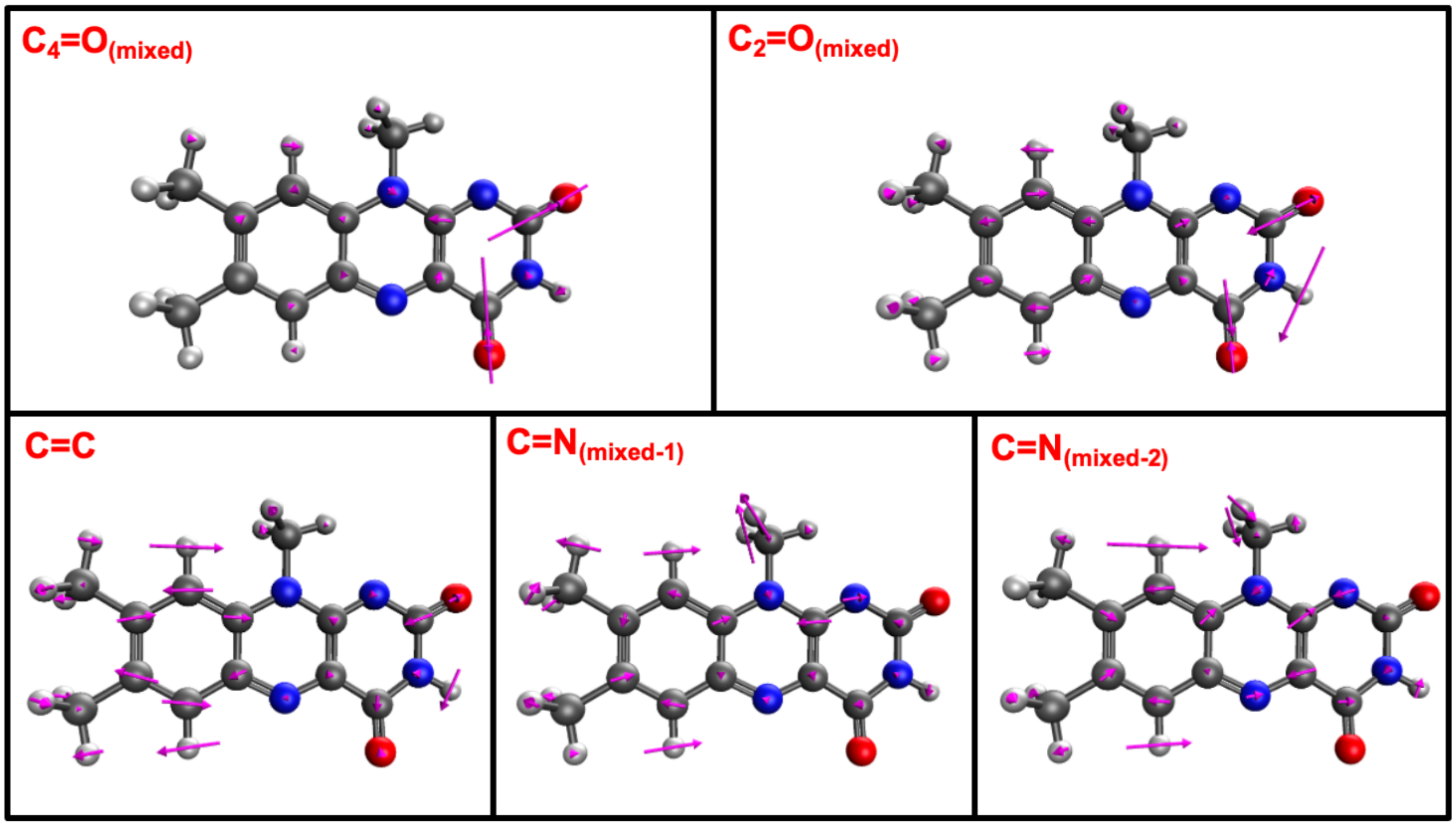

Figure S3: Normal modes computed with the B3LYP/6-31+G*/IEF-PCM model for triplet LF (n,π*).

Table S1: B3LYP/6-31+G*/PCM computed vibrational frequencies (cm^{-1}) after scaling by a 0.989 factor followed by % contribution of specific modes based on VEDA analysis for triplet FMN (π,π*).

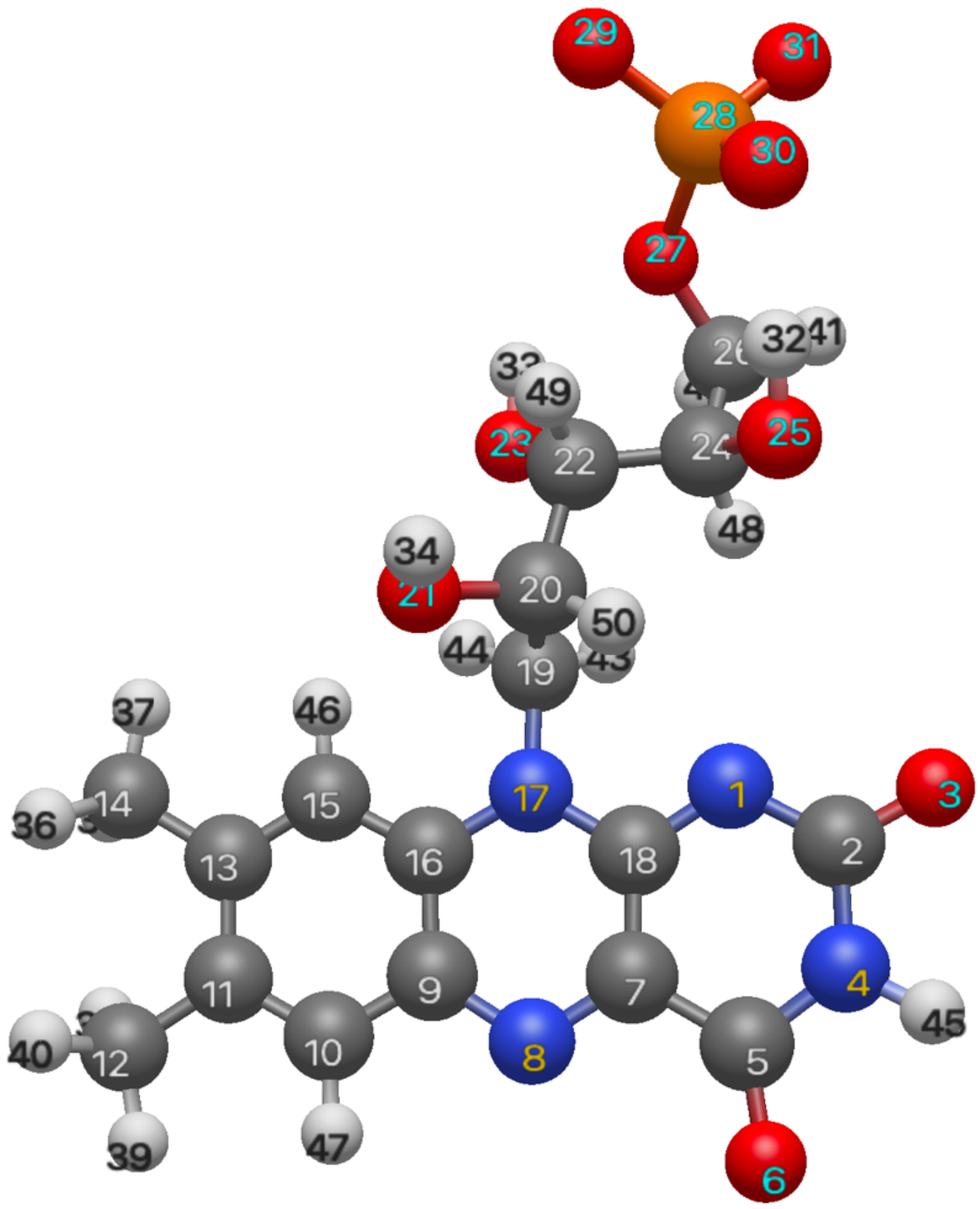

Frequency (cm^{-1})	Vibrational Mode	Atoms involved in internal coordinates	Contribution (%)
1627	OC stretch	3 – 2	50
		6 – 5	
1605	OC stretch	3 – 2	-53
		6 – 5	
	HNC bend	45 – 4 – 5	19
1527	HCC bend	46 – 15 – 13	30
		47 – 10 – 11	
1501	NC stretch	1 – 2	-12
		1 – 18	
		8 – 7	
		8 – 9	
	HCH bend	35 – 14 – 37	-17
		38 – 12 – 40	
		39 – 12 – 38	
		40 – 12 – 39	
1477	HCH bend	42 – 26 – 41	-20
		44 – 19 – 43	
	HCH bend	42 – 26 – 41	-19
		44 – 19 – 43	
1425	CC stretch	9 – 10	10
		11 – 10	

		15 – 13	
		16 – 15	
	HCH bend	36 – 14 – 35	-10
	NCC bend	1 – 18 – 7	-10
	CNC bend	2 – 1 – 18	
	CNC bend	7 – 8 – 9	
	NCC bend	8 – 9 – 10	
1365	NC stretch	4 – 2	12
		4 – 5	
		17 – 16	
		17 – 18	
	HCN bend	43 – 19 – 17	17

Table S2: B3LYP/6-31+G*/PCM computed vibrational frequencies (cm^{-1}) after scaling by a 0.990 factor followed by % contribution of specific modes based on VEDA analysis for singlet FMN.

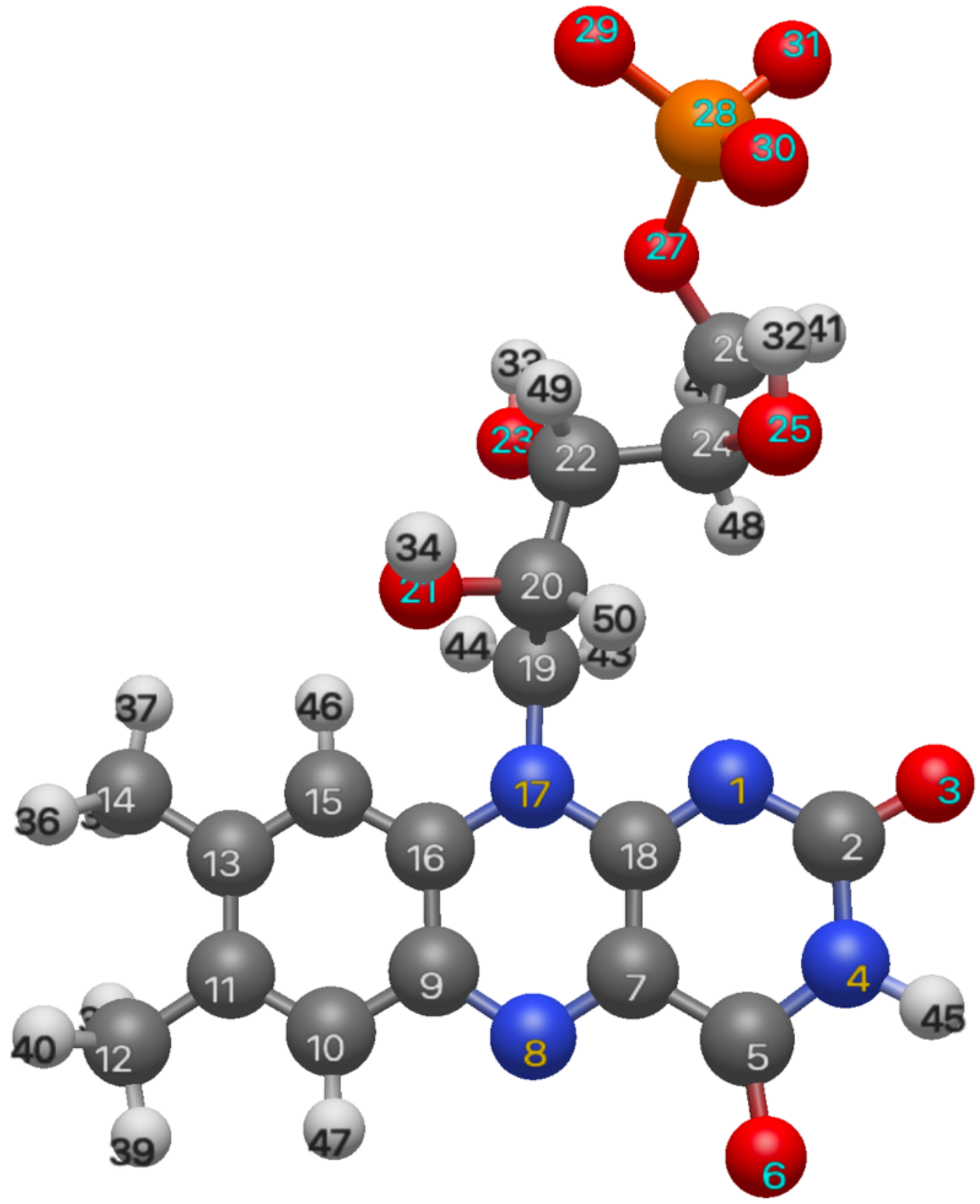

Frequency (cm^{-1})	Vibrational Mode	Atoms involved in internal coordinates	Contribution (%)
1549	NC stretch	1 – 18	-10
		8 – 7	
	CC stretch	10 – 9	12
		11 – 10	
		13 – 15	
		16 – 15	
	NC stretch	4 – 2	-12
		4 – 5	
		17 – 16	
		17 – 18	
1590	NC stretch	1 – 18	-22
		8 – 7	
	CC stretch	9 – 16	21
1673	OC stretch	3 – 2	-54
		6 – 5	
	OC stretch	3 – 2	12
		6 – 5	
1717	OC stretch	3 – 2	13
		6 – 5	
	OC stretch	3 – 2	61
		6 – 5	

Table S3: Comparison of selected bond lengths (in Å) within the isoalloxazine ring of LF and FMN optimized in the singlet and triplet electronic states at the B3LYP/6-31+G* level of theory. Both gas phase and IEF-PCM model calculations are shown.

Molecule	C_{10a}=N_1	C_{4a}=N_5	C_{4a}=C_{10a}	C_2=O	C_4=O
**LF gas singlet**	1.31	1.30	1.46	1.22	1.22
**FMN gas singlet**	1.32	1.32	1.45	1.23	1.23
**LF PCM singlet**	1.32	1.31	1.45	1.23	1.23
**FMN PCM singlet**	1.32	1.31	1.45	1.23	1.23
**LF gas triplet**	1.33	1.36	1.42	1.23	1.22
**FMN gas triplet**	1.34	1.36	1.43	1.24	1.24
**LF PCM triplet**	1.32	1.35	1.42	1.24	1.24
**FMN PCM triplet**	1.34	1.36	1.42	1.25	1.25

Table S4: B3LYP/6-31+G*/PCM computed vibrational frequencies (cm⁻¹) after scaling by a 0.989 factor followed by % contribution of specific modes based on VEDA analysis for triplet LF.

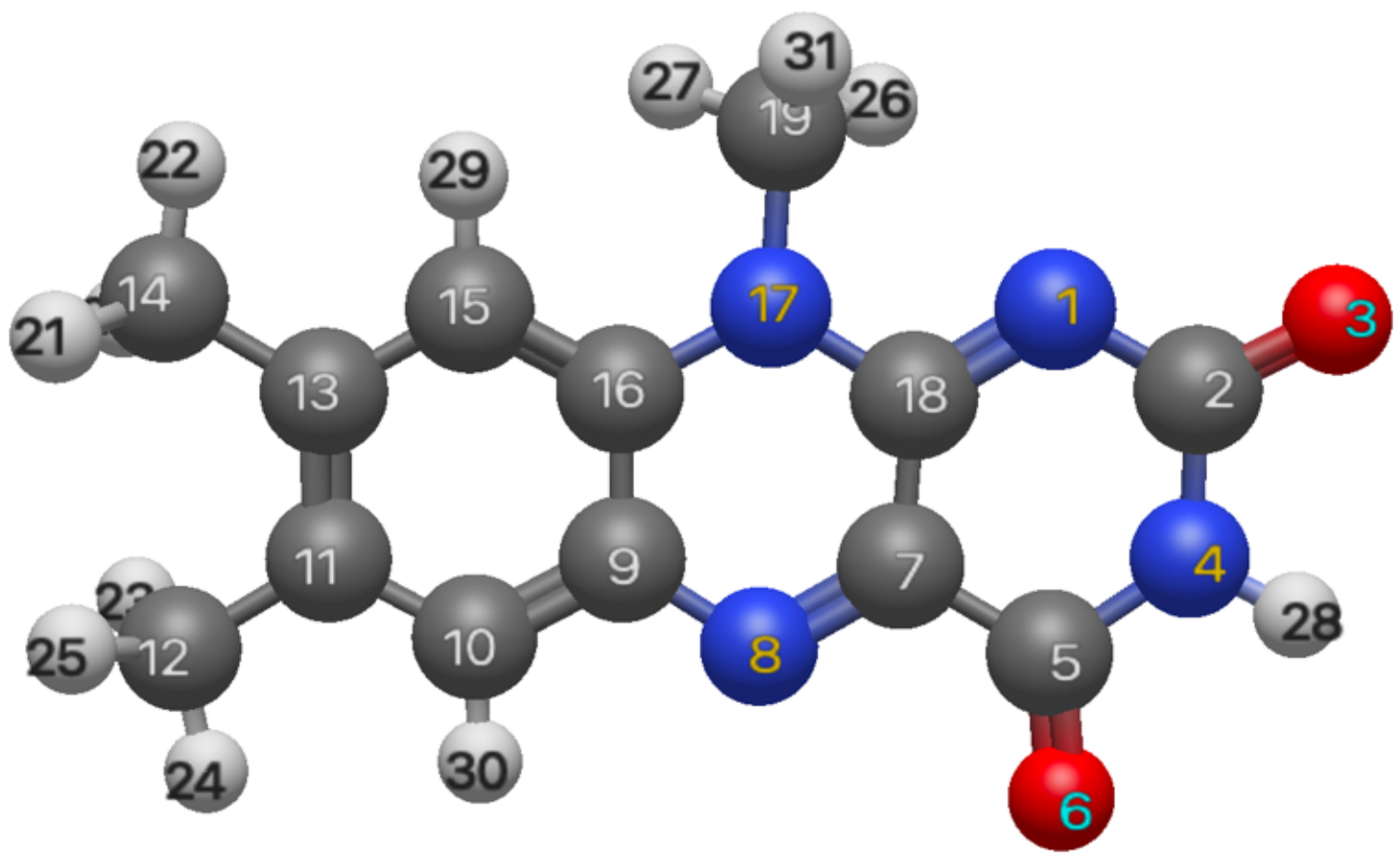

Frequency (cm^{-1})	Vibrational Mode	Atoms involved in internal coordinates	Contribution (%)
1652	OC stretch	3 – 2	77
		6 – 5	
1628	OC stretch	3 – 2	-57
		6 – 5	
	CC stretch	9 – 10	10
		16 – 15	
		11 – 10	
		15 – 13	
	HNC bend	28 – 4 – 5	13
1614	OC stretch	3 – 2	-12
		6 – 5	
	CC stretch	9 – 10	-50
		16 – 15	
		11 – 10	
		15 – 13	
	HCC bend	30 – 10 – 11	13
		29 – 15 – 13	
1524	NC stretch	1 – 2	17
		1 – 18	
		8 – 7	
		8 – 9	
	HCC bend	30 – 10 – 11	13
		29 – 15 – 13	
1497	NC stretch	1 – 2	-26
		1 – 18	
		8 – 7	
		8 – 9	
	HCC bend	30 – 10 – 11	22
		29 – 15 – 13	

B3LYP/6-31+G* Optimized Structure of singlet LF in gas-phase

C	3.766990	-0.635165	-0.000066
N	3.739130	0.780209	0.000082
C	2.629627	1.602658	0.000156
C	1.340624	0.835340	-0.000007
N	2.558765	-1.304447	-0.000066
C	1.440952	-0.624614	-0.000004
N	0.221687	1.496041	-0.000107
C	-0.950936	0.790660	-0.000126
N	0.238004	-1.309792	-0.000005
C	-0.971812	-0.629012	-0.000001
C	-2.169862	1.500640	-0.000200
C	-3.395527	0.855302	-0.000099
C	-3.410920	-0.570214	0.000118
C	-2.214706	-1.286461	0.000174
O	4.848147	-1.201786	-0.000175
H	4.646463	1.236890	0.000163
O	2.707668	2.819384	0.000338
C	0.252070	-2.778711	-0.000126
H	-2.105138	2.585107	-0.000298
C	-4.681793	1.646602	-0.000249
C	-4.722052	-1.316858	0.000316
H	-2.258807	-2.369360	0.000446
H	1.290160	-3.102547	-0.000691
H	-0.255239	-3.153684	-0.894272
H	-0.254252	-3.153867	0.894514
H	-5.295807	1.423347	-0.882344
H	-4.477922	2.721352	0.000396
H	-5.296603	1.422440	0.881055
H	-4.565777	-2.399542	0.000868
H	-5.326196	-1.063441	-0.880320
H	-5.326434	-1.062564	0.880534

B3LYP/6-31+G* Optimized Structure of singlet LF in PCM

C	3.754453	-0.629817	-0.000243
N	3.734663	0.781001	-0.000106
C	2.622534	1.589446	0.000083
C	1.342269	0.826756	0.000097
N	2.567956	-1.304323	-0.000200
C	1.434351	-0.623714	0.000010
N	0.219366	1.494310	0.000175
C	-0.948343	0.797622	0.000140
N	0.241265	-1.305410	0.000216
C	-0.967555	-0.626358	0.000142
C	-2.172337	1.507729	0.000054
C	-3.391876	0.858626	-0.000044
C	-3.403087	-0.572924	-0.000022
C	-2.207985	-1.287447	0.000030
O	4.852704	-1.194038	-0.000369
H	4.640954	1.240897	-0.000121
O	2.701711	2.815225	0.000252
C	0.241759	-2.778835	0.000329

H	-2.116778	2.592569	0.000052
C	-4.681658	1.641339	-0.000325
C	-4.711574	-1.319344	-0.000089
H	-2.251763	-2.369359	-0.000060
H	1.272962	-3.117568	0.001466
H	-0.268709	-3.142625	-0.894703
H	-0.270647	-3.142435	0.894301
H	-5.291983	1.408621	-0.881926
H	-4.484516	2.716980	-0.000035
H	-5.292692	1.408251	0.880711
H	-4.553472	-2.400954	-0.000110
H	-5.313543	-1.060687	-0.880353
H	-5.313588	-1.060727	0.880151

B3LYP/6-31+G* Optimized Structure of singlet FMN in gas-phase

N	-2.403617	-2.514206	-0.277845
C	-3.382675	-3.459308	-0.161554
O	-3.200880	-4.672650	-0.266119
N	-4.703790	-3.012521	0.096009
C	-5.147993	-1.708192	0.203722
O	-6.334287	-1.446174	0.402806
C	-4.057642	-0.715582	0.050840
N	-4.360098	0.566584	0.118334
C	-3.345759	1.461790	-0.018436
C	-3.646122	2.844234	0.051609
C	-2.670238	3.818224	-0.065933
C	-3.032215	5.283314	0.018005
C	-1.318541	3.407637	-0.254113
C	-0.208983	4.427987	-0.354861
C	-1.000386	2.053279	-0.331849
C	-1.993398	1.060200	-0.227102
N	-1.714441	-0.298066	-0.329740
C	-2.721843	-1.233972	-0.177782
C	-0.339192	-0.756391	-0.599338
C	0.545737	-0.926803	0.682071
O	0.061824	-0.124315	1.762718
C	2.005445	-0.552178	0.394142
O	1.986338	0.867605	0.140481
C	2.711438	-1.380002	-0.723063
O	3.246888	-2.556820	-0.147824
C	3.766193	-0.521810	-1.472699
O	4.416887	0.437160	-0.656421
P	5.822457	-0.088251	0.283834
O	6.077651	1.144970	1.143633
O	5.258732	-1.323426	1.040321
O	6.868097	-0.445254	-0.776502
H	4.066629	-2.246527	0.358616
H	2.950785	1.081397	-0.000334
H	0.580300	0.700255	1.724002
H	-0.351079	5.097548	-1.215465
H	-0.165771	5.067259	0.537951
H	0.763782	3.940507	-0.462270

H	-2.743471	5.828705	-0.891362
H	-4.111572	5.410370	0.156155
H	-2.526247	5.781506	0.856675
H	4.495902	-1.197727	-1.939345
H	3.255915	0.036718	-2.274608
H	-0.425364	-1.716468	-1.105383
H	0.111841	-0.035815	-1.281709
H	-5.404557	-3.740429	0.183594
H	0.043756	1.775488	-0.427689
H	-4.689340	3.107519	0.208401
H	1.983311	-1.718843	-1.477382
H	2.585427	-0.736671	1.307394
H	0.483542	-1.966901	1.011266

B3LYP/6-31+G* Optimized Structure of singlet FMN in PCM

N	-0.964309	-2.333672	-0.369265
C	-1.219347	-3.670003	-0.254473
O	-0.353347	-4.538641	-0.395578
N	-2.533814	-4.088327	0.043881
C	-3.634585	-3.283564	0.220806
O	-4.747389	-3.738802	0.474007
C	-3.329446	-1.833502	0.072013
N	-4.303634	-0.972444	0.198567
C	-4.020537	0.349909	0.063772
C	-5.077332	1.283635	0.192974
C	-4.870080	2.642519	0.071031
C	-6.015591	3.614583	0.206437
C	-3.538494	3.105311	-0.179371
C	-3.262048	4.581581	-0.302087
C	-2.487706	2.202583	-0.304804
C	-2.697750	0.815324	-0.201073
N	-1.687896	-0.128325	-0.349864
C	-1.955576	-1.470693	-0.216764
C	-0.304952	0.264736	-0.705703
C	0.617165	0.370790	0.513338
O	0.098815	1.427316	1.335386
C	2.074840	0.623324	0.100654
O	2.103505	1.647655	-0.898831
C	2.802079	-0.681121	-0.338386
O	3.176412	-1.412899	0.826744
C	3.994221	-0.380069	-1.263297
O	4.740695	0.773765	-0.862706
P	6.033664	0.603179	0.262686
O	6.433502	2.065793	0.505030
O	5.383780	-0.094034	1.494350
O	7.084406	-0.269912	-0.446521
H	4.005428	-0.965465	1.180921
H	3.052627	1.841577	-1.031788
H	0.529092	1.379756	2.204536
H	-3.835088	5.022702	-1.127548
H	-3.558498	5.117140	0.608609
H	-2.201307	4.774900	-0.482225

H	-6.140019	4.216882	-0.702212
H	-6.954906	3.086442	0.393140
H	-5.851127	4.316557	1.033554
H	4.639001	-1.266361	-1.316174
H	3.621151	-0.176122	-2.273773
H	0.070242	-0.499282	-1.384619
H	-0.341947	1.208198	-1.242971
H	-2.675864	-5.091162	0.127573
H	-1.491828	2.595148	-0.454418
H	-6.067517	0.883728	0.392079
H	2.118169	-1.331485	-0.899286
H	2.603590	0.977646	0.996379
H	0.589210	-0.573102	1.069253

B3LYP/6-31+G* Optimized Structure of triplet LF in gas-phase

C	-3.778213	-0.635400	-0.000158
N	-3.764145	0.751314	-0.000042
C	-2.634298	1.579971	0.000011
C	-1.360028	0.846283	0.000009
N	-2.545056	-1.289327	-0.000056
C	-1.430819	-0.569663	-0.000013
N	-0.199623	1.561388	0.000074
C	0.935506	0.836077	0.000043
N	-0.225196	-1.302655	0.000005
C	0.975348	-0.617591	-0.000018
C	2.179873	1.512718	0.000077
C	3.396288	0.849194	0.000030
C	3.420912	-0.587774	-0.000058
C	2.215675	-1.282453	-0.000076
O	-4.829756	-1.267432	0.000031
H	-4.665599	1.218511	0.000019
O	-2.751616	2.798197	0.000127
C	-0.246225	-2.766768	0.000007
H	2.137826	2.597725	0.000133
C	4.683191	1.631422	0.000088
C	4.728443	-1.337319	-0.000121
H	2.243919	-2.366178	-0.000141
H	-1.282202	-3.095129	0.000101
H	0.261237	-3.146264	0.893478
H	0.261065	-3.146277	-0.893558
H	5.295257	1.395434	0.880684
H	4.490725	2.707597	0.000022
H	5.295412	1.395344	-0.880371
H	4.563195	-2.418944	-0.000164
H	5.337206	-1.092624	0.880608
H	5.337165	-1.092551	-0.880858

B3LYP/6-31+G* Optimized Structure of triplet LF in PCM

C	-3.763290	-0.644300	-0.000095
N	-3.765325	0.738922	0.000033
C	-2.641513	1.564062	-0.000024
C	-1.365379	0.850251	-0.000101

N	-2.536984	-1.290723	-0.000132
C	-1.428141	-0.570976	-0.000141
N	-0.219403	1.570937	-0.000108
C	0.937060	0.845423	-0.000063
N	-0.214451	-1.295808	-0.000206
C	0.975141	-0.604717	-0.000068
C	2.165167	1.517122	-0.000006
C	3.396270	0.848160	0.000090
C	3.423501	-0.586376	0.000138
C	2.220306	-1.275239	0.000053
O	-4.822307	-1.281092	0.000152
H	-4.670579	1.200151	0.000195
O	-2.772860	2.792602	0.000190
C	-0.224082	-2.764808	-0.000326
H	2.139852	2.602644	-0.000028
C	4.674057	1.633882	0.000167
C	4.730828	-1.334483	0.000281
H	2.243823	-2.357975	0.000094
H	-1.255752	-3.101599	-0.000647
H	0.284065	-3.135915	0.894079
H	0.284584	-3.135749	-0.894498
H	5.285554	1.390323	0.879195
H	4.482449	2.709527	-0.000012
H	5.285835	1.390067	-0.878590
H	4.563666	-2.415004	0.000382
H	5.335669	-1.084834	0.881392
H	5.335758	-1.085016	-0.880823

B3LYP/6-31+G* Optimized Structure of triplet FMN in gas-phase

N	-1.090585	-2.377055	-0.300174
C	-1.381158	-3.707406	-0.243018
O	-0.549708	-4.616233	-0.398755
N	-2.716572	-4.061876	0.012836
C	-3.811932	-3.207190	0.158208
O	-4.941601	-3.685696	0.349199
C	-3.460129	-1.797489	0.053517
N	-4.447968	-0.866675	0.135550
C	-4.070539	0.431785	0.029523
C	-5.066136	1.440952	0.092216
C	-4.777131	2.799646	0.010185
C	-5.891057	3.821630	0.083463
C	-3.428709	3.200456	-0.130241
C	-3.048645	4.662897	-0.191675
C	-2.424236	2.221318	-0.197980
C	-2.711521	0.852142	-0.141920
N	-1.722341	-0.143011	-0.248225
C	-2.080811	-1.492746	-0.159180
C	-0.344274	0.191235	-0.639121
C	0.631880	0.386319	0.526799
O	0.218224	1.557719	1.259662
C	2.094054	0.516853	0.046739
O	2.123229	1.142579	-1.240721

C	2.837915	-0.848914	0.090716
O	3.194907	-1.128318	1.442032
C	4.036627	-0.891562	-0.869556
O	4.776485	0.344522	-0.925868
P	6.056381	0.628353	0.104692
O	6.450588	2.087974	-0.187311
O	5.586480	0.288596	1.511492
O	7.262438	-0.215986	-0.389619
H	4.018735	-0.619957	1.639745
H	3.064955	1.323610	-1.417896
H	0.754829	1.595627	2.066417
H	-3.510045	5.178344	-1.048757
H	-3.363973	5.213449	0.708342
H	-1.963274	4.779290	-0.283241
H	-5.933145	4.453354	-0.816697
H	-6.864505	3.328486	0.188125
H	-5.771038	4.504472	0.938116
H	4.693035	-1.725234	-0.593128
H	3.677456	-1.060099	-1.890094
H	0.020788	-0.635355	-1.248439
H	-0.363912	1.087863	-1.259023
H	-2.915329	-5.054252	0.060959
H	-1.393503	2.548741	-0.244392
H	-6.091342	1.096439	0.211666
H	2.158092	-1.656952	-0.210101
H	2.616449	1.162766	0.768008
H	0.568758	-0.486394	1.187172

B3LYP/6-31+G* Optimized Structure of triplet FMN in PCM

N	-0.790398	-2.242575	-0.349467
C	-0.910847	-3.587502	-0.241244
O	0.047981	-4.381301	-0.375223
N	-2.174156	-4.113336	0.038724
C	-3.353111	-3.390953	0.202672
O	-4.417993	-3.999731	0.438775
C	-3.195242	-1.956966	0.070330
N	-4.294606	-1.159859	0.190396
C	-4.089431	0.182747	0.065237
C	-5.198289	1.057623	0.179812
C	-5.087944	2.440248	0.081232
C	-6.312391	3.317209	0.209994
C	-3.805014	3.003842	-0.132953
C	-3.618972	4.500344	-0.227700
C	-2.693904	2.157301	-0.249581
C	-2.805270	0.762953	-0.172457
N	-1.702126	-0.105623	-0.319457
C	-1.882306	-1.476816	-0.195860
C	-0.374160	0.390555	-0.708716
C	0.590901	0.570700	0.470018
O	0.131857	1.697699	1.235172
C	2.045159	0.762686	0.008487
O	2.060867	1.676941	-1.093766

C	2.731676	-0.590880	-0.323732
O	3.012785	-1.285983	0.892319
C	3.969509	-0.415611	-1.211257
O	4.772161	0.736457	-0.848913
P	5.966460	0.649753	0.278358
O	6.379209	2.121444	0.515590
O	5.455900	-0.140720	1.470842
O	7.249239	0.074377	-0.390735
H	3.844382	-0.907294	1.264886
H	2.985810	1.950015	-1.221688
H	0.575644	1.682944	2.098667
H	-4.195603	4.929723	-1.058365
H	-3.956865	5.011519	0.684020
H	-2.566782	4.758257	-0.384517
H	-6.471737	3.926210	-0.689812
H	-7.211799	2.714065	0.369837
H	-6.223810	4.017450	1.051328
H	4.572254	-1.329738	-1.188712
H	3.662798	-0.232448	-2.243502
H	0.046722	-0.333225	-1.405891
H	-0.493501	1.332413	-1.241024
H	-2.243765	-5.122319	0.122050
H	-1.720783	2.615341	-0.371758
H	-6.168977	0.598275	0.353540
H	2.040770	-1.236049	-0.878178
H	2.599793	1.199267	0.851293
H	0.559328	-0.329298	1.092889
